# Long-Term Results of Tinnitus Retraining Therapy in Patients Who Failed to Complete the Program

**DOI:** 10.3390/audiolres11010001

**Published:** 2021-01-12

**Authors:** Ruggero Lapenna, Egisto Molini, Laura Cipriani, Maria Rita Del Zompo, Giorgia Giommetti, Mario Faralli, Giampietro Ricci

**Affiliations:** Section of Otolaryngology-Head and Neck Surgery, Department of Medicine, University of Perugia, 06123 Perugia, Italy; ruggerolapenna@gmail.com (R.L.); egimolini@libero.it (E.M.); mariaritadelzompo@gmail.com (M.R.D.Z.); giorgia.giommetti@gmail.com (G.G.); mario.far@hotmail.it (M.F.); ricci1501@hotmail.com (G.R.)

**Keywords:** tinnitus, TRT, follow-up, missing

## Abstract

**Purpose:** We aimed to evaluate the results of Tinnitus Retraining Therapy (TRT) in patients who did not complete the program. **Methods:** We divided 90 patients who failed to complete the TRT program were into 3 groups: 36 patients who only completed the first phase of the TRT program (Missing group; M), 34 patients who attended counselling for less than 6 months (Noncompliant group; NC) and 20 patients who attended counselling for more than 6 months but did not complete the TRT program (Compliant group; C). The Tinnitus Handicap Inventory (THI), tinnitus Visual Analogue Scales (VAS) and a questionnaire regarding the reasons for dropout were obtained through a telephone survey. **Results:** Telephonic THI and VAS scores were significantly lower than the initial scores in the M and C groups but not in the NC group. Patients who were unsure about the effectiveness of TRT were prevalent in the NC group, and the poorest long-term THI results were registered in those patients. **Conclusions:** A fundamental cause of very poor TRT results was when patients were unsure about TRT. On the other hand, a single counselling session could be effective in reducing tinnitus annoyance in patients who accepted the TRT approach and trusted its efficacy.

## 1. Introduction

Subjective tinnitus is a sound perception which is exclusively dependent on the activity of the nervous system, without any type of mechanical or vibratory activity of the cochlea and independent of any external stimulation. In 1% of cases, tinnitus becomes a disabling condition based on the level of activation of the limbic and autonomic nervous systems and requires treatment [1]. Jastreboff and Jastreboff devised a neurophysiological model of tinnitus, postulating the involvement of the limbic and autonomic nervous systems in the onset of tinnitus. They also developed a specific treatment strategy: Tinnitus Retraining Therapy (TRT) [2].

TRT consists of counselling/teaching sessions and sound therapy that create two kinds of habituation: Habituation to perception and habituation of reaction. Habituation is reached by reducing the strength of the tinnitus signal and showing its benign character, providing an explanation about its origin and mechanisms and pointing out that patients have “a proper reaction to an improper stimulus” [3].

Patients reach habituation to their bothersome tinnitus in 12–18 months, consistent with the quantitative and qualitative definition of “bothersome tinnitus” given by Molini et al. [4]. The results from many tinnitus treatment centres have shown that TRT causes noticeable improvements in 74–84% of patients with any type of tinnitus, according to definite outcome measures [2,5,6,7,8,9]. The first improvement appears 3 months after starting TRT [4,10] and increases 6 months after starting TRT [11]. Although its effectiveness has been widely documented in the literature, there have been few studies on the efficacy of TRT in noncompliant patients [12,13]. In particular, no study has correlated the results of an incomplete TRT program with the timing and reasons for abandoning the program.

The aim of this study was to evaluate the actual situation in patients experiencing chronic tinnitus who did not return to our clinic for a proper follow-up, with particular reference to the timing and reasons for their dropout.

## 2. Methods

This is a retrospective case–control study on a population of patients experiencing chronic disabling tinnitus who started TRT in the period between January 2011 and December 2018 at the Department of Otolaryngology of University of Perugia (Italy).

Patients had to satisfy the following inclusion criteria:

1. Age over 18 years;
2. Disabling tinnitus for more than 6 months;
3. No tinnitus treatments for at least 1 year before starting or during the TRT program, in terms of specific pharmacologic, physical or nonconventional therapies (i.e., acupuncture);
4. No pathologic features at brain MRI scan and Doppler ultrasonography of the epi-aortic vessels;
5. Category 1 tinnitus patients according to Jastreboff and Hazell’s classification [14] (tinnitus is an important problem, without subjective hearing loss);
6. No diagnosed associated psychiatric disorders precluding full participation or follow-up;
7. No cognitive impairment which could influence adherence to the clinical protocol;
8. Completion of at least the first phase of the TRT program (directive counselling, tinnitus psychoacoustic measurements and participation in sound therapy);
9. Noncompletion of the entire TRT program, namely the therapist did not define the case as successfully closed after 12–18 months of follow-up;
10. No previous history of otologic diseases or migrainous vertigo;
11. No involvement in litigation or seeking monetary compensation for their tinnitus.

All patients were contacted for a telephone survey using an independent researcher naive to TRT who administered the tinnitus Visual Analogue Scale (VAS), with ratings from 0 to 10 for Loudness (L), Annoyance (A) and Effect on life (E); the Italian validated version of the Tinnitus Handicap Inventory (THI) [15,16]; and our telephone questionnaire (Table 1).

We divided the patients into 3 groups according to the timing of their dropout from the TRT program:

1. Missing group (M; *n* = 36): Patients who completed the first phase of the TRT program but did not return for the second counselling appointment.
2. Noncompliant group (NC; *n* = 34): Patients who attended counselling appointments for less than 6 months.
3. Compliant group (C; *n* = 20): Patients who attended counselling appointments for more than 6 months but did not complete the TRT program of 12–18 months duration.

A group of 28 control cases (CC) was included, composed of patients who satisfactorily completed the TRT program. The whole study sample was also divided into 3 groups according to their answers to question number 3 (A, B, C) in Table 1.

The authors assert that all procedures contributing to this work comply with the ethical standards of the relevant national and institutional guidelines on human experimentation and with the Helsinki Declaration of 1975, as revised in 2008. All patients gave their informed consent.

### Statistical Analysis

The main parameters of the investigations were the average values shown as mean ± standard deviation (SD) for THI and for tinnitus VAS L, VAS A and VAS E, registered at the beginning of TRT and during the telephone survey, and the type of answers to the questionnaire.

The comparison of continuous data that was normally distributed (*p* < 0.05; Shapiro–Wilk test) between more than 2 groups was conducted with the parametric 1-way ANOVA with Tukey’s post-hoc test (to avoid type I and II errors). The comparison of continuous data that was not normally distributed (*p* > 0.05; Shapiro–Wilk test) between more than 2 groups was conducted with the nonparametric Kruskal–Wallis test, with a post-hoc pairwise comparison using the Mann–Whitney *U*-test. The comparison of continuous data between paired groups was conducted with a parametric paired *t*-test or with the Wilcoxon test depending on the normality of the distribution. The comparison of categorical data (distribution of the answers to the questionnaire among the groups) was conducted with the Chi-squared test, with a logistic regression using the Odds Ratio (OR) in the case of relevant answers in selected cases. *p* values <0.05 were considered to be statistically significant.

## 3. Results

In the present study, 90 patients (mean PTA 20.48 ± 11.84 dB HL for 0.5–1–2 kHz) satisfied the inclusion criteria and were included: 36 were assigned to group M (age 54 ± 13.49; M:F = 16:20), 34 to group NC (age 52.59 ± 8.39; M:F = 22:12) and 20 to group C (age 51.9 ± 8.85; M:F = 10:10). The groups were not significantly different in terms of age (*p* > 0.05; one-way ANOVA) or sex (*p* > 0.05; Pearson Chi-squared test).

Mean time elapsed from the last TRT appointment to the telephone survey was 32.66 ± 10.85 months in group M, 29.71 ± 12.30 months in group NC and 27.9 ± 11.90 months in group C, with no statistically significant difference between the groups (*p* > 0.05; one-way ANOVA).

The control group was composed of 28 subjects matched for age (56.08 ± 11.80), sex (M:F = 18:10) and time elapsed from the end of TRT to the telephone survey (31.71 ± 15.78 months).

The results for THI and VAS L, A and E, registered at the beginning of TRT (I) and during the telephone survey (T), and associated statistical analyses are reported in Table 2.

**Figure 1:**
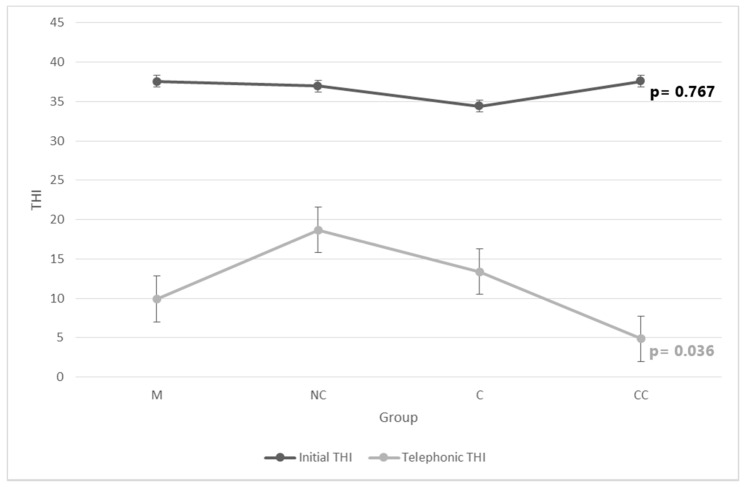
Initial and telephonic THI values in each study group with their relative error bars and significance.

The initial THI score and initial and telephonic VAS scores were not significantly different among groups (see Figure 1 and Table 2). Post-hoc pairwise comparison showed a statistically significant difference only in the telephonic VAS A between NC and CC groups (*p* < 0.05; Mann–Whitney *U*-test).

On the other hand, the telephonic THI score differed significantly among groups (*p* = 0.036; Kruskal–Wallis test) (Figure 1). Post-hoc pairwise comparison showed a statistically significant difference in the telephonic THI only between the NC and CC groups (*p* < 0.05; Mann–Whitney *U*-test).

Table 1 shows the distribution of answers to the telephonic questionnaire with associated statistical analysis. A significant difference between groups was detected in the distribution of answers to questions no. 3 (*p* = 0.023) and no. 5 (*p* = 0.07; Pearson’s Chi-squared test).

Considering the distribution of answers to question no. 2, we obtained the following telephonic THI scores: 37.75 ± 15.87 in patients considering tinnitus “an insurmountable problem,” 12.61 ± 9.92 in patients considering tinnitus “a lifelong partner” and 2.71 ± 5.58 in patients considering tinnitus “no longer a problem,” with a statistically significant difference (*p* = 0.000; Kruskal–Wallis test).

The study sample was then divided into three groups based on the answers to question no. 3: Group A (40 patients who were unsure about the effectiveness of TRT), group B (20 patients who were cured after the first counselling) and group C (30 patients who dropped out for other reasons). Table 3 reports the initial and telephonic THI and VAS scores with corresponding statistical analysis.

**Figure 2:**
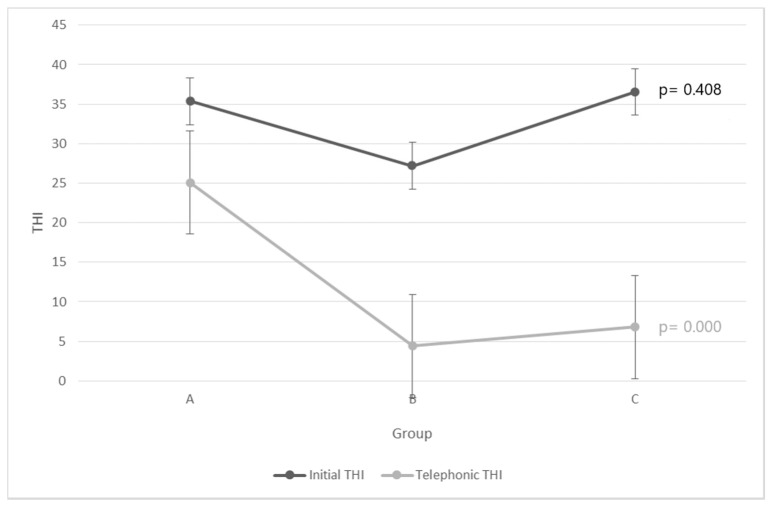
Initial and telephonic THI values in patients divided according to their answers to question no. 3 of the questionnaire (Table 1) with their relative error bars and significance.

Initial THI and telephonic VAS scores did not differ among groups (Figure 2) or among all of the categories of VAS at the beginning of TRT (Table 3). Post-hoc pairwise comparison showed a statistically significant difference only in VAS E between groups B and C (*p* < 0.05; Mann–Whitney *U*-test).

On the other hand, the telephonic THI and VAS scores were different among groups. In particular, the reasons for dropout had a significant effect on telephonic THI and VAS scores (Table 3).

Post-hoc pairwise comparison showed significant differences (*p* < 0.05; Mann–Whitney *U*-test) in the telephonic THI between groups A and B and groups A and C, in the telephonic VAS L between groups A and B and groups A and C, in the telephonic VAS A between groups A and B and groups A and C, and in the telephonic VAS E between groups A and B.

## 4. Discussion

Jastreboff based TRT on a neurophysiological model. The distress associated with tinnitus arises from abnormal subconscious nonauditory mechanisms, mediated primarily by the limbic and autonomic nervous systems. The goal of TRT is habituation to a dangerous signal, the tinnitus, eliminating the reaction to it and minimizing its perception [1].

Counselling aims to reclassify tinnitus as a neutral signal, while sound therapy aims to weaken tinnitus-related neuronal activity [3]. When the TRT protocol is closely followed, the typical success rate ranges from 74% to 83.7% improvement [2,5,6,7,8,9]. However, the literature lacks randomized trials on the effectiveness of TRT [17,18,19], so there is insufficient evidence that TRT is superior to other treatments [20,21].

Although many factors affecting TRT results have been investigated [22], there is little information about the effectiveness of TRT on those patients who have not fully completed the TRT program.

In a study using a telephone survey, Han et al. pointed out that treatment outcomes, evaluated with THI and tinnitus VAS, were better in patients lost to follow-up than in good TRT followers (more than 3 months of therapy) [13]. Conversely, Forti et al. demonstrated that tinnitus was no longer a problem for only a small percentage (26%) of patients who did not return for follow-up after receiving the initial counselling and sound therapy [12]. These contrasting results, and the absence of definite data about predictors of adherence to TRT and dropout, motivated this study. To the best of our knowledge, this is the first paper in which patients who did not complete TRT were evaluated according to the timing and reasons for their dropout with the aim of examining their long-term outcomes.

According to our results, baseline tinnitus VAS L, A and E scores and scores of the initial THI showed moderate-severe handicap [15], but the initial values of THI and VAS did not influence the timing of TRT dropout. This is in contrast to previous observations by Molini et al. that more severe tinnitus is more likely to be associated with better outcomes [4]. Worse values of the telephonic THI score in patients who defined their tinnitus as ‘an insurmountable problem’ confirmed that THI and tinnitus VAS are brief, easily administered and psychometrically robust measures to evaluate the severity of tinnitus and its impact on daily life.

The main result of this study is that THI and tinnitus VAS E scores obtained during the telephonic survey were significantly lower than the initial values in all of the study groups, apart from the NC group.

Counselling sessions without additional sound therapy have been reported to be as effective as TRT in reducing the annoyance and impact of tinnitus [23]. We might expect to find an overall improvement in tinnitus which is directly proportional to the number of counselling sessions attended. However, we observed comparable results between group M and the control group.

A fundamental cause of very poor TRT results was when patients were unsure about TRT as an effective therapy for disabling tinnitus. Fewer patients from group M reported that they were unsure about the effectiveness of the therapeutic program compared with the patients from the NC group (OR = 0.875). For this reason, the efficacy of a single counselling session can be considered to be a valid explanation for the significant improvement in THI in the M group. This possibility is supported by a lower perception of tinnitus as disabling (OR = 0.47), the less frequent utilization of sound therapy (OR= 0.58) and not feeling the need to turn to alternative tinnitus therapies (OR = 0.4).

We can then formulate three different hypotheses to explain the poorest results in the NC group: (1) Poor intrinsic/extrinsic motivation, (2) incorrect expectations about the results and (3) incorrect expectations about the role of the counsellor. Adherence to TRT and its outcomes is positively associated with two kinds of motivation, intrinsic and extrinsic, as described for psychotherapy [24]. The first is self-regulated and the subjective perception of benefit reinforces it. The counsellor can implement the second [25].

Patients in the NC group would have decided to attend further TRT sessions (for less than 6 months) with poor intrinsic motivation, even though they had not fully accepted the program. They might have discontinued treatment, as they had perceived themselves to be not responding. On the other hand, poor extrinsic motivation could be assumed in these patients. To avoid this, a TRT clinic should be appropriately organized to provide effective counselling and sound therapy when treating tinnitus patients [26]. Our TRT team comprises a senior counsellor and three “junior counsellors.” The first is an audiologist/otolaryngologist with 20 years of experience, competent in the diagnosis and treatment of tinnitus through TRT and who attended Jastreboff’s courses and received direct training. Three otolaryngologists complement the team who have undergone a 6-month training program using Jastreboff’s protocol with direct assistance from the senior counsellor.

The interactions we have with patients create expectations that can affect the treatment based on their understanding of tinnitus [27]. According to this second hypothesis, patients from group NC would have dropped out from TRT, as they had not achieved the expected improvement.

The counsellor should inform patients about the possibility of the transient worsening of tinnitus and about the significant time commitment (at least 6 months) required to achieve and maintain success [11] to provide realistic expectations about the course of treatment. Many patients have an incorrect expectation about the role of the counsellor, as they feel that the achievement of improvements should depend only on the counsellor and that their role in the treatment is entirely passive.

If the patient continues to focus on the tinnitus and its negative associations, its perception can be exacerbated. Effective control of these maladaptive emotional reactions is an important component of tinnitus management [28,29] based on collaboration between patient and counsellor.

This study gives robust evidence for the view that poor motivation and incorrect expectations can lead to unsatisfactory results for TRT. On the other hand, a single counselling session could be efficacious, in particular, in cases where the explanation of the tinnitus mechanism is sufficient to obtain relief from the annoyance. However, this last observation is limited by the retrospective nature of this study, and new prospective randomized studies are required on a larger cohort of patients to establish the shorter effective length of a successful TRT program.

The last important consideration from this study is that, once obtained, the improvement in THI score persists for a long time (the average time elapsed between the last TRT session and the telephone survey was about 30 months in our study groups). This means that TRT has a significant long-term effect and the habituation process continues and persists after the conclusion of TRT, in agreement with the literature [6,9,30].

In addition to the retrospective nature of this work, its main limitations were the lack of a control group who did not undergo any therapy and the absence of a specific analysis based on some data that were not considered or missing (for example, sex-specific).

## 5. Conclusions

An early dropout from TRT and poorer results are expected in patients who were unsure about the effectiveness of the proposed therapeutic program. In fact, poor motivation in following the TRT program and incorrect expectations about its outcomes can lead to unsatisfactory results. A single counselling session could be effective in reducing tinnitus annoyance in the study sample. However, further prospective studies, including a control group of patients undergoing no therapies at all, could clarify the potential role of TRT alone in the facilitation of the habituation process and the lower number of counselling sessions needed to obtain and maintain the result.

## Figures and Tables

**Table 1:** Telephone survey questionnaire and distribution of answers with associated statistical analysis (Pearson’s Chi-squared test).

		M	NC	C	CC	*p*
1. Do you still have ear tinnitus?	Yes	28 (78%)	30 (88%)	16 (80%)	26 (93%)	0.33
	No	8 (22%)	4 (12%)	4 (20%)	2 (7%)	
2. Tinnitus is now:	(A) “an insurmountable problem”	4 (11%)	8 (24%)	4 (20%)	2 (7%)	0.59
	(B) “a lifelong partner”	20 (56%)	16 (47%)	10 (50%)	14 (50%)	
	(C) “no longer a problem”	12 (33%)	10 (29%)	6 (30%)	12 (43%)	
3. What was the reason for TRT dropout?	(A) I did not accept the effectiveness of TRT	18 (50%)	18 (53%)	4 (20%)	-	**0.023**
	(B) My tinnitus was cured after the first counselling session	4 (11%)	10 (29%)	6 (30%)	-	
	(C) Other reasons (for example: illness, distance, occupation)	14 (39%)	6 (18%)	10 (50%)	-	
4. Do you still use sound therapy?	Yes	4 (11%)	6 (18%)	8 (40%)	10 (36%)	0.29
	No	32 (89%)	28 (82%)	12 (60%)	18 (64%)	
5. Did you later choose other kinds of therapy for tinnitus?	Yes	4 (11%)	8 (24%)	0 (0%)	0 (0%)	**0.007**
	No	32 (89%)	26 (76%)	20 (100%)	28 (100%)	

M, missing; NC, noncompliant; C, compliant; CC, control cases; *p* values in bold are statistically significant (*p* < 0.05).

**Table 2:** Results from THI and VAS in the different groups at the beginning of TRT and during the telephone survey and associated statistical analysis.

			M (*n* = 36)	NC (*n* = 34)	C (*n* = 20)	CC (*n* = 28)	*p*
THI		I	37.57 ± 20.53	36.94 ± 18.69	34.4 ± 17.15	37.57 ± 20.53	*p_A_* = 0.767
		T	9.89 ± 10.94	18.70 ± 14.01	13.40 ± 19.16	4.86 ± 8.79	***p_K_* = 0.036**
*p*			***p_W_* = 0.000**	*p_W_* = 0.051	***p_W_* = 0.012**	***p_W_* = 0.001**	
VAS	L	I	6.06 ± 1.53	5.82 ± 2.26	6.28 ± 1.39	6.12 ± 1.30	*p_K_* = 0.706
		T	4.19 ± 3.16	5.18 ± 2.92	4.45 ± 3.12	4.14 ± 2.50	*p_K_* = 0.659
	*p*		***p_W_* = 0.006**	*p_W_* = 0.313	*p_W_* = 0.185	***p_W_* = 0.014**	
	A	I	6.67 ± 2.09	6.76 ± 2.43	7 ± 2.23	7.23 ± 1.64	*p_K_* = 0.992
		T	3.77 ± 2.90	5.08 ± 2.79	4.15 ± 2.83	2.46 ± 2.24	*p_K_* = 0.058
	*p*		***p_W_* = 0.001**	***p_W_* = 0.034**	***p_W_* = 0.01**	***p_W_* = 0.001**	
	E	I	4.37 ± 3.57	5.03 ± 3.04	6.05 ± 2.67	4.42 ± 2.48	*p_K_* = 0.603
		T	2.72 ± 2.19	2.85 ± 3.15	2.85 ± 3.15	1.43 ± 1.97	*p_K_* = 0.243
	*p*		***p_W_* = 0.006**	*p_W_* = 0.12	***p_W_* = 0.036**	***p_W_* = 0.005**	

M, missing group; NC, noncompliant group; C, compliant group; CC, control group; THI, Tinnitus Handicap Inventory; VAS, Visual Analogue Scale; L, loudness; A, annoyance; E, effect on life; I, initial; T, telephone survey; *p_A_*, *p* value from one-way ANOVA; *p_K_*, *p* value from Kruskal–Wallis test; *p_W_*, *p* value from Wilcoxon test. *p* values in bold are statistically significant (*p* < 0.05).

**Table 3:** Results from THI and VAS at the beginning of TRT and during the telephone survey according to the answers to question no. 3 of the questionnaire (Table 1) and associated statistical analysis.

		A	B	C	*p*
THI	I	35.35 ± 16.48	27.2 ± 20.09	36.53 ± 18.57	*p_A_* = 0.408
	T	25.09 ± 17.21	4.4 ± 5.15	6.8 ± 6.18	***p_K_* = 0.000**
*p*		***p_T_* = 0.029**	***p_T_* = 0.002**	***p_W_* = 0.000**	
VAS L	I	6.58 ± 1.94	5 ± 1.78	6.4 ± 1.72	*p_A_* = 0.083
	T	6 ± 2.97	2.9 ± 2.78	3.93 ± 2.55	***p_K_* = 0.009**
*p*		*p_T_* = 0.337	***p_W_* = 0.041**	***p_T_* = 0.008**	
VAS A	I	6.95 ± 1.81	5.85 ± 2.73	7.26 ± 2.19	*p_A_* = 0.266
	T	5.775 ± 2.68	2.55 ± 2.49	3.7 ± 2.45	***p_K_* = 0.002**
*p*		*p_W_* = 0.061	***p_W_* = 0.006**	***p_T_* = 0.000**	
VAS E	I	5.35 ± 3.20	4 ± 2.91	6.13 ± 2.58	*p_K_* = 0.161
	T	4.475 ± 2.96	0.95 ± 1.23	2.56 ± 2.06	***p_K_* = 0.005**
*p*		*p_W_* = 0.234	***p_W_* = 0.022**	***p_T_* = 0.000**	

Group A, patients who were unsure about the effectiveness of TRT; group B, patients who were cured after the first counselling session; group C, patients who dropped out for other reasons; THI, Tinnitus Handicap Inventory; VAS, Visual Analogue Scale; L, loudness; A, annoyance; E, effect on life; I, initial; T, telephonic; *p_A_*, *p* value from one-way ANOVA; *p_K_*, *p* value from Kruskal–Wallis test; *p_W_*, *p* value from Wilcoxon test. *p_T_*, *p* value from paired *t*-test. *p* values in bold are statistically significant (*p* < 0.05).

## Data Availability

The data presented in this study are available on request from the corresponding author. The data are not publicly available due to the presence of sensitive information in the database.
